# Identifying long-term patterns and predictors of concurrent psychotropic medicine use in residential aged care using group-based multi-trajectory modelling: the ‘MEDTRAC-Psychotropics’ longitudinal cohort study

**DOI:** 10.1007/s41999-025-01171-6

**Published:** 2025-02-25

**Authors:** Nasir Wabe, Isabelle Meulenbroeks, Desiree Chantelle Firempong, Rachel Urwin, Andrea Timothy, Magdalena Z. Raban, Virginia Mumford, Johanna Westbrook

**Affiliations:** Centre for Health Systems and Safety Research, Australian Institute of Health Innovation, Macquarie University, Level 6, 75 Talavera Road, North Ryde, NSW 2122 Australia

**Keywords:** Aged care, Nursing homes, Medication use, Group-based multi-trajectory modelling, Psychotropics

## Abstract

**Purpose:**

Psychotropic medicines are commonly used in residential aged care facilities (RACFs) despite notable safety concerns. No prior studies have examined the longitudinal concurrent use of psychotropic medicines. We aimed to identify trajectories of concurrent use of three psychotropic medication classes over time and determine predictors of trajectory group membership for residents with and without dementia.

**Methods:**

A retrospective longitudinal cohort study including 30 RACFs in Sydney, Australia. The study participants included 2837 newly admitted permanent residents (*n* = 1344 with dementia) aged ≥ 65 years. We monitored weekly exposure to three psychotropic classes—antidepressants, antipsychotics, and anxiolytics/hypnotics—over three years. We used group-based multi-trajectory modelling to identify concurrent psychotropic medicine use.

**Results:**

At baseline, 38.5%, 19.6%, and 16.7% of residents with dementia received antidepressants, antipsychotics, and anxiolytics/hypnotics, respectively, compared to 32.8%, 7.1%, and 16.5% in residents without dementia. The concurrent use of multiple psychotropic classes occurred in 23.3% of non-dementia and 31.6% of dementia cohorts. The model identified 6-group and 4-group trajectories as the optimal fit for dementia and non-dementia cohorts, respectively. Psychotropic use trajectories mostly remained stable over time in non-dementia cohorts, while dementia cohorts showed more diverse and fluctuating use. Multinomial logistic regressions identified eleven predictors of trajectory membership in dementia and nine in non-dementia cohorts.

**Conclusion:**

One in three residents with dementia and one in five without dementia concurrently use multiple psychotropics often for extended periods, which may put residents at risk. Further research should assess the appropriateness of such use and consider strategies for improving health outcomes.

## Key summary points

**Aim:**

Our study aimed to identify trajectories of concurrent use of three psychotropic medication classes over time and identify predictors of trajectory group membership for residents with and without dementia.

**Findings:**

We conducted a retrospective longitudinal cohort study among residents in 30 RACFs in New South Wales, Australia and found that one in three residents with dementia and one in five residents without dementia concurrently use multiple psychotropics often for extended periods, which may put residents at risk.

**Message:**

Considering a concerning high rate of concurrent use of multiple psychotropic medicines in RACFs, it is imperative to assess its appropriateness and consider further research and strategies for improved health outcomes.

## Introduction

Psychotropic medicines are widely used in residential aged care facilities (RACFs). In Australia, nearly two-thirds of older adults in RACFs receive psychotropic medicines regularly [1]. While important in the management of mental health conditions, the judicious use of psychotropic medicines in RACF settings is necessary due to associated risks in older adults, including impaired cognition, heightened stroke and pneumonia risk, and a twofold increase in hip fracture risk [2–4]. The risks associated with this drug class increase with the concurrent use of multiple psychotropics [5].

International guidelines often restrict recommendations for psychotropic use to specific populations, emphasising regular medication reviews to taper usage and facilitate discontinuation when feasible. For example, the American Geriatrics Association recommends that benzodiazepines, a class of psychotropic medicine, be used sparingly to manage sleep disorders in older adults [6]. In Australia, it is recommended that the use of benzodiazepines be reviewed every two to four weeks [7]. Other psychotropic medicines such as antidepressants for the management of major depression and antipsychotics for the management of conditions such as schizophrenia can be clinically appropriate in older adults [6]. However, the use of antidepressants and antipsychotics should be reviewed every six and three months, respectively [7].

Although indications for the use of psychotropic medicines in older adults are limited, they are widely used in RAC settings often for their sedative effects in managing behavioural and psychological symptoms of dementia (BPSD) such as agitation, wandering, and aggression [8]. However, evidence supporting their effectiveness in managing BPSD is limited [5]. Internationally, psychotropic use in RACFs varies, with countries consistently reporting high proportions of residents using these medicines for extended periods, exceeding recommended guidelines [9].

There is a dearth of research examining the longitudinal patterns of psychotropics use in RACF settings. In Australia, the recently introduced National Aged Care Quality Indicator Program mandates quarterly reporting of antipsychotic use [10] in order to monitor the proportion of residents receiving antipsychotics. However, these data are cross-sectional aggregated data, which fail to provide insights into how usage varies over time for individuals. Further, while residents may simultaneously use different psychotropic medication classes over time, no studies have explored the concurrent use of multiple psychotropic medications over time. Recognising the complexity of these usage patterns, it is imperative to employ a longitudinal design that allows for a more comprehensive understanding of how psychotropic medicines are utilised over extended periods following RACF admission. This approach ensures a nuanced assessment of the dynamic and evolving nature of psychotropic medication use among RACF residents.

To better understand the longitudinal patterns of the use of multiple psychotropic medicine classes in RACFs, we conducted a large-scale multi-centre study utilising group-based multi-trajectory modelling. This approach enables the simultaneous modelling of psychotropic classes, identifying latent clusters of individuals with similar trajectories over time. In this MEDTRAC (MEDicine use Trajectories in Residential Aged Care)-Psychotropic study, we aimed to (1) identify concurrent developmental trajectories of the use of three psychotropic medicine classes over three years in RACFs and (2) determine factors associated with trajectory group membership. The analysis is separated by dementia status as this is an important confounding factor in the use of antipsychotics. The MEDTRAC-Psychotropic study was a part of the National Aged Care Medication Roundtable project funded by the Australian National Health and Medical Research Council Partnership Grant (APP2006957).

## Methods

### Study setting

We conducted a retrospective longitudinal cohort study, utilising electronic records from 30 RACFs in Sydney, New South Wales, Australia. The RACFs belong to two different aged care providers, with 24 facilities from Provider A and 6 from Provider B. The study period was from 1 January 2019 to 30 September 2022. We followed the RECORD (REporting of studies Conducted using Observational Routinely collected health Data) statement to structure this paper [11]. This study was reviewed and approved by the Macquarie University Human Research Ethics Committee (ref: 520231126749629).

### Participants

The inclusion criteria were permanent residents aged 65 years or older, residents who entered the facilities on or after 1 January 1 2019, and stayed at the facility for a minimum of 30 days to allow for the completion of at least four follow-up data points. We specifically excluded residents who were already residing in the RACF as of 1 January 1 2019. This was because our study focused on examining the trajectory of psychotropic medicines use for newly admitted individuals over time. Those already present in the facility at the study’s commencement may have been exposed to psychotropic medications, potentially leading to different trajectories compared to newly admitted residents.

### Data source

We utilised routinely collected, de-identified electronic data obtained from aged care providers, specifically the *resident profile* and *medication administration databases*. The *resident profile database* included demographic details such as age, sex, care provider information, and health condition (e.g. dementia, Parkinson’s disease, or diabetes). Health conditions in our dataset were recorded as free-text entries, which were processed using a previously developed health macro to identify specific conditions [12]. These diagnoses were primarily documented at the time of the resident’s entry into residential aged care. For example, psychosis-related diagnoses were identified using free-text terms such as schizophrenia, paranoia, and psychoses. The *medication administration database* contained details of the daily medications administered to each resident. We applied the World Health Organization’s Anatomical Therapeutic Classification (ATC) codes to identify relevant medication classes.

### Psychotropic medicines

We examined three psychotropic medicine classes commonly prescribed in Australia [13]: (1) antipsychotics (ATC code N05A excluding lithium [N05AN] and prochlorperazine [N05AB04]); (2) anxiolytics (N05B) and sedatives and hypnotics (N05C) (referred to as ‘anxiolytics/hypnotics’ *hereafter*); and (3) antidepressants (N06A, N06CA). For each psychotropic, we assessed the weekly usage (Y/N) from the time of RACF admission to a maximum of three years (156 data points). We presented the results separately by dementia status at baseline due to the distinct characteristics observed in individuals with dementia and the safety concerns associated with the use of psychotropics in this population [4, 14–16].

### Statistical analysis

We used a group-based multi-trajectory modelling approach to determine the concurrent developmental trajectories of the use of the three psychotropic classes over three years, developing separate models by dementia status. Multi-trajectory modelling is an extension of the group-based trajectory modelling (GBTM) methodology [17], originally designed to model a single indicator of interest. Multi-trajectory modelling allows for the simultaneous analysis of multiple indicators (the three psychotropic classes in this case), identifying latent clusters of individuals following similar trajectories over time. In instances where individuals have a follow-up duration of less than three years, it assumes that missing data are randomly distributed and uses available data to impute any missing values during subsequent follow-up intervals [17]. We employed established model selection and performance metrics to determine the optimal number of multi-trajectory groups, including Bayesian Information Criterion (BIC), Akaike Information Criterion (AIC), group size (minimum of 5%), average posterior probability (> 0.7 for all groups), relative entropy (≥ 0.8 indicating high classification certainty), and odds of correct classification (≥ 5 for all groups) [18–22]. Lower BIC and AIC values indicate better model fit. Furthermore, we evaluated the robustness of our selected number of multi-trajectory groups by conducting fivefold cross-validation on random data subsets, examining the reliability of both model fit statistics and group sizes. Once the trajectory groups were identified, the naming of the groups was based on the visual inspection of the patterns in the trajectory figures.

After identifying the multi-trajectory groups for both non-dementia and dementia cohorts, multinomial logistic regression was used to determine predictors of multi-trajectory group membership. The analysis considered demographics (age, sex, provider), year of admission, baseline health conditions (Table 1), and baseline medication use (ATC level 1) defined as medicines administered to a resident during the first week of admission to the RACF. We first performed with univariate analysis, choosing variables with *P* ≤ 0.2 for subsequent multivariate multinomial regression with backward hierarchical selection to identify independent predictors of the multi-trajectory group membership. We estimated the strength of association using a relative risk ratio (RRR) with a 95% confidence interval (CI) which quantifies the likelihood of belonging to a specific group in relation to the reference group for a one-unit increase in the predictor of interest, while holding all other variables in the model constant. All *P*-values were two tailed, and statistical significance was set at P < 0.05. The analysis was performed using Stata version 18 (StataCorp LP, College Station, TX).

**Table 1:** Baseline participant characteristics by dementia status

Variables, *n* (%) unless otherwise specified	Dementia		Total (*n* = 2837)
	Yes (*n* = 1344)	No (*n* = 1493)	
Female	814 (60.6)	937 (62.8)	1751 (61.7)
Age in year, median (IQR)	86.0 (81.0–90.0)	87.0 (81.0–92.0)	86.0 (81.0–91.0)
Year of admission			
2019	381 (28.3)	392 (26.3)	773 (27.2)
2020	313 (23.3)	302 (20.2)	615 (21.7)
2021	401 (29.8)	422 (28.3)	823 (29.0)
2022 (Jan–Sep)	249 (18.5)	377 (25.3)	626 (22.1)
Health conditions			
Circulatory conditions, any	1188 (88.4)	1364 (91.4)	2552 (90.0)
Cerebrovascular accident	322 (24.0)	369 (24.7)	691 (24.4)
Endocrine, any	526 (39.1)	656 (43.9)	1182 (41.7)
Diabetes	365 (27.2)	498 (33.4)	863 (30.4)
Thyroid	161 (12.0)	159 (10.6)	320 (11.3)
Chronic respiratory	214 (15.9)	357 (23.9)	571 (20.1)
Cancer	375 (27.9)	542 (36.3)	917 (32.3)
Parkinson disease	123 (9.2)	147 (9.8)	270 (9.5)
Depression, mood, and affective disorders	576 (42.9)	621 (41.6)	1197 (42.2)
Anxiety and stress-related disorders	381 (28.3)	449 (30.1)	830 (29.3)
Psychosis^a^	113 (8.4)	61 (4.1)	174 (6.1)
Peptic Ulcer/Gastro-Oesophageal Reflux Disease	400 (29.8)	500 (33.5)	900 (31.7)
Renal disease	245 (18.2)	359 (24.0)	604 (21.3)
Arthritis	707 (52.6)	821 (55.0)	1528 (53.9)
Osteoporosis	399 (29.7)	403 (27.0)	802 (28.3)
Gout	113 (8.4)	195 (13.1)	308 (10.9)
Fracture	405 (30.1)	473 (31.7)	878 (30.9)
Hearing impairment	250 (18.6)	313 (21.0)	563 (19.8)
Visual impairment	189 (14.1)	242 (16.2)	431 (15.2)
Polypharmacy without PRN (9 or more medicines)	340 (25.3)	498 (33.4)	838 (29.5)
Medications (ATC level 1), *n* (%)			
Alimentary tract and metabolism	1146 (85.3)	1368 (91.6)	2514 (88.6)
Antineoplastic and immunomodulators	55 (4.1)	72 (4.8)	127 (4.5)
Blood and blood-forming organs	761 (56.6%)	924 (61.9)	1685 (59.4)
Cardiovascular system	991 (73.7)	1203 (80.6)	2194 (77.3)
Genitourinary system and sex hormones	163 (12.1)	209 (14.0)	372 (13.1)
Musculoskeletal system	180 (13.4)	299 (20.0)	479 (16.9)
Nervous system	1080 (80.4)	1194 (80.0)	2274 (80.2)
Respiratory system	187 (13.9)	386 (25.9)	573 (20.2)
Sensory organs	281 (20.9)	427 (28.6)	708 (25.0)
Systemic hormonal preparations	261 (19.4)	371 (24.8)	632 (22.3)

^a^Based on free-text entries containing the terms schizophrenia, paranoia, or psychoses

*ATC* anatomical therapeutic classification, *PRN* ‘pro re nata’

## Results

### Participants

The study sample included 2,837 residents (47.4%, *n* = 1344 having a dementia diagnosis at baseline). Of the total sample, 61.7% were female, the median age was 86 years, and 29.5% experienced polypharmacy (9 or more regular medicines) at baseline. Table 1 shows the comparison of participant characteristics by dementia status at baseline. Age distribution and the prevalence of several health conditions significantly varied by dementia status. For instance, the proportion of individuals with diabetes was 27.2% in the dementia cohort, and 33.4% in the non-dementia cohort (*P* < 0.001). Except for medicines for the nervous system, and genitourinary system and sex hormones, all other medicine classes showed significant differences between dementia and non-dementia cohorts. Individuals with dementia received fewer medications compared to their counterparts (*P* < 0.001).

### Trends in aggregate psychotropic medicines use

**Fig. 1:**
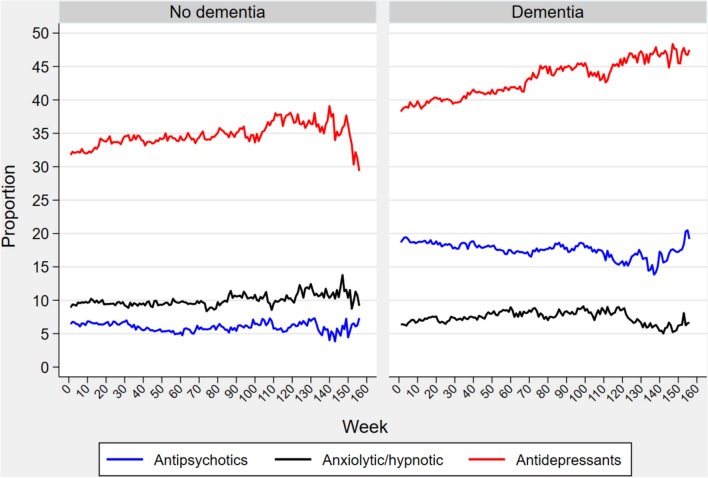
Aggregated weekly psychotropic medication usage by dementia status. The y-axis represents the proportion of residents using respective medications

Figure 1 presents the aggregate trends in the use of psychotropic medicines over a 3-year period. Antidepressants were the most prevalent psychotropics in both cohorts. In the non-dementia cohort, baseline prevalence was 32.8% and increased to 39.1%, while in the dementia cohort, it was 38.5% at baseline, consistently rising over time to reach 48.4%. The use of antipsychotics and antidepressants was higher in the dementia cohort compared to the non-dementia cohort. Conversely, the use of anxiolytics/hypnotics was relatively consistent between the two cohorts, with a slight decline observed towards the end of year three in the dementia cohort.

### Multi-trajectory modelling of psychotropic use by dementia status

**Fig. 2:**
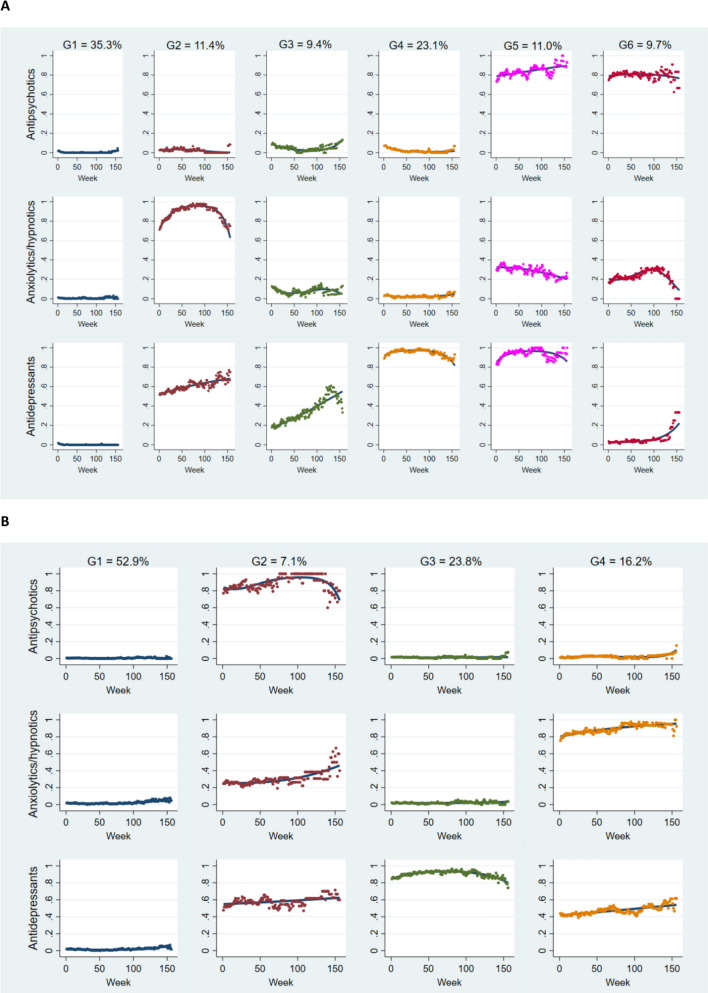
Psychotropic use multi-trajectories by dementia status during the first three years after RACF entry. **A** Dementia; **B** no dementia. The solid lines indicate estimated probabilities, while the dot symbols denote the observed probabilities. The y-axis indicates the probability of using respective medications

Multi-trajectory GBTM modelling revealed a 4-group model as the optimal fit for the non-dementia cohort, while a 6-group model best suited the dementia cohort (Fig. 2). The model fit statistics and performance parameters were excellent in both cohorts, including average posterior probability ≥ 0.98, odds of correct classification > 500, and relative entropy > 0.98 for all groups.

In the dementia cohort, the patterns of psychotropic use exhibited greater complexity, consisting of a mix of various patterns (Fig. 2A). There were no major fluctuations in the patterns of psychotropic use in non-dementia cohorts (Fig. 2B). Table 2 presents the characteristics of the identified groups for both cohorts. The predominant group in both cohorts consisted of psychotropic non-users, constituting more than half of the non-dementia residents at 52.9%, whereas in the dementia cohort, this group accounted for a comparatively lower proportion at 35.3%. The findings indicate that concurrent use of multiple psychotropic classes occurred in 23.3% of non-dementia (Groups 2 and 4 combined) and 32.1% of dementia (Groups 2, 5, and 6) cohorts during the study period (Table 2).

**Table 2:** The identified groups and their characteristics

Group	Size, *n* (%)	Group characteristics
No dementia (*n* = 1493)		
G1-Psychotropic non-users	790 (52.9)	The largest group with negligible use of all three psychotropics across 3 years
G2-Antipsychotic users with moderate other psychotropics use	106 (7.1)	High antipsychotic use slightly declining after year 2 with moderate use of anxiolytics/hypnotics and antidepressants
G3-Antidepressant users	356 (23.8)	High antidepressant use with negligible use of other psychotropics across 3 years
G4-Anxiolytics/hypnotics users with moderate antidepressants	241 (16.2)	High anxiolytics/hypnotics use and moderate use of antidepressants across 3 years
Dementia(*n* = 1344)		
G1-Psychotropic non-users	475 (35.3)	The largest with negligible use of all three psychotropics
G2-Anxiolytics/hypnotics users with gradually rising antidepressant use	153 (11.4)	High initial anxiolytics/hypnotics use, followed by a decrease, with moderate but gradually increasing antidepressant use
G3-Gradually increasing antidepressant users	126 (9.4)	Low but increasing antidepressant use with minimal exposure to other psychotropics
G4-Antidepressant users	311 (23.1)	High antidepressant use and negligible use of other psychotropics across 3 years
G5-Antipsychotic and antidepressant users with low anxiolytics/hypnotics use	148 (11.0)	High concurrent usage of antipsychotics and antidepressants with low anxiolytics/hypnotics use
G6-Antipsychotic users with low anxiolytics/hypnotics	131 (9.7)	High antipsychotic usage, low anxiolytics/hypnotics use, and negligible antidepressant use

### Factors associated with multi-trajectory membership

Tables 3 and 4 present findings of multinomial logistic regressions using Group 1 (psychotropic non-users) as a reference group. In the non-dementia cohort, nine variables were associated with at least one trajectory group. Diagnoses related to mental health, the use of nervous system medications or being on polypharmacy, all increased the likelihood of belonging to one of the three groups (Groups 2–4) in comparison to the reference group, as anticipated. For example, for individuals having depression, mood, and affective disorders at baseline, the relative risk of belonging to Group 3 (antidepressant users with negligible other psychotropics use) over Group 1 increased by a factor of 18.51 after adjusting for variables in the model (RRR 18.51; 95% CI 12.40–27.65). Interestingly, increasing age, having diabetes, or receiving medications for blood and blood-forming organs or cardiovascular system were associated with a reduced likelihood of belonging to Group 2 (antipsychotic users) versus Group 1 (Table 3).

**Table 3:** Multinomial logistic regressions showing predictors of psychotropic use multi-trajectory membership in individuals without dementia

Variable	Group 2	Group 3	Group 4
	RRR (95% CI)	RRR (95% CI)	RRR (95% CI)
Sex	1.22 (0.64–2.32)	1.15 (0.77–1.74)	1.22 (0.75–2.00)
Age at admission	**0.93** (**0.90–0.97**)	0.98 (0.96–1.01)	1.00 (0.97–1.03)
Provider B vs. Provider A	1.29 (0.77–2.16)	1.11 (0.48–2.55)	1.09 (0.53–2.22)
Polypharmacy (9 or more medicines)	**2.07** (**1.24–3.44**)	1.52 (0.99–2.34)	**2.01 (1.36–2.97)**
Health conditions^a^			
Cerebrovascular accident	1.06 (0.59–1.90)	1.17 (0.87–1.57)	0.83 (0.52–1.30)
Diabetes	**0.61** (**0.39–0.95**)	0.85 (0.59–1.22)	0.75 (0.54–1.03)
Thyroid	0.67 (0.35–1.29)	1.20 (0.67–2.15)	1.17 (0.61–2.25)
Chronic respiratory	0.56 (0.29–1.08)	0.91 (0.57–1.47)	1.41 (0.85–2.35)
Cancer	0.75 (0.44–1.29)	0.98 (0.69–1.41)	0.81 (0.55–1.18)
Parkinson’s disease	1.52 (0.80–2.88)	1.42 (0.89–2.26)	1.40 (0.85–2.33)
Depression, mood, and affective disorders	**6.35** (**3.64–11.09**)	**18.51 (12.40–27.65)**	**3.30 (2.15–5.04)**
Anxiety and stress-related disorders	**1.96** (**1.20–3.19**)	**1.38 (1.03–1.86)**	**2.07 (1.45–2.96)**
Peptic ulcer/Gastro-Oesophageal Reflux Disease	1.12 (0.60–2.09)	1.03 (0.78–1.36)	0.83 (0.56–1.23)
Renal disease	**2.15** (**1.24–3.74**)	**1.51 (1.08–2.1)**	**1.52 (1.09–2.13)**
Arthritis	0.80 (0.47–1.38)	0.95 (0.71–1.28)	0.83 (0.59–1.17)
Osteoporosis	0.81 (0.48–1.37)	0.86 (0.59–1.26)	0.90 (0.64–1.27)
Gout	1.21 (0.63–2.31)	1.25 (0.73–2.15)	1.09 (0.64–1.84)
Fracture	0.93 (0.56–1.56)	0.89 (0.62–1.27)	0.99 (0.69–1.40)
Hearing impairment	0.70 (0.44–1.10)	0.92 (0.56–1.51)	0.94 (0.60–1.48)
Visual impairment	0.90 (0.48–1.68)	0.85 (0.59–1.23)	0.79 (0.55–1.13)
ATC medication classes (Level 1)			
Alimentary tract and metabolism	1.05 (0.29–3.80)	1.49 (0.71–3.13)	1.13 (0.57–2.23)
Antineoplastic and immunomodulators	1.19 (0.39–3.60)	1.10 (0.42–2.9)	0.76 (0.27–2.12)
Blood and blood-forming organs	**0.51** (**0.32–0.82**)	0.96 (0.67–1.37)	1.05 (0.66–1.69)
Cardiovascular system	**0.36** (**0.23–0.57**)	**0.57 (0.38–0.85)**	**0.48 (0.29–0.80)**
Genitourinary system and sex hormones	1.36 (0.98–1.89)	0.98 (0.64–1.49)	1.14 (0.69–1.87)
Musculoskeletal system	0.50 (0.25–1.01)	0.83 (0.54–1.27)	0.72 (0.50–1.04)
Nervous system	**6.24** (**2.32–16.79**)	**4.81 (2.50–9.28)**	**9.28 (4.22–20.41)**
Respiratory system	1.46 (0.83–2.55)	0.79 (0.51–1.24)	0.86 (0.60–1.24)
Sensory organs	0.71 (0.43–1.17)	0.78 (0.54–1.13)	0.90 (0.61–1.34)
Systemic hormonal preparations	1.20 (0.70–2.05)	1.14 (0.80–1.64)	0.82 (0.52 – 1.31)

*Group 1* (psychotropic non-users) as a reference category. *P* < 0.05 is indicated in bold

*Group 2*: Antipsychotic users with moderate exposure to anxiolytics/hypnotics and antidepressants; *Group 3*: Antidepressant users with negligible exposure to other psychotropics; Group 4: Anxiolytics/hypnotics users with moderate use of antidepressants

^a^Psychosis was not included in the model due to the small sample size in each group

**Table 4:** Multinomial logistic regressions showing predictors of psychotropic use multi-trajectory membership in individuals with dementia

Variable	Group 2	Group 3	Group 4	Group 5	Group 6
	RRR (95% CI)	RRR (95% CI)	RRR (95% CI)	RRR (95% CI)	**RRR (95% CI)**
Sex	0.61 (0.36–1.03)	0.93 (0.52–1.67)	1.10 (0.74–1.65)	0.87 (0.42–1.78)	0.65 (0.36–1.16)
Age at admission	0.99 (0.96–1.03)	1.01 (0.97–1.04)	0.99 (0.96–1.02)	**0.94 (0.91–0.97)**	**0.97 (0.94–0.99)**
Provider B vs. Provider A	**2.02 (1.23–3.30)**	1.10 (0.73–1.65)	1.28 (0.90–1.81)	1.84 (0.84–4.05)	1.49 (0.8–2.74)
Polypharmacy without PRN	**3.53 (2.25–5.54)**	1.09 (0.52–2.28)	**1.94 (1.27–2.95)**	**2.68 (1.68–4.28)**	1.36 (0.86–2.15)
Health conditions^a^					
Cerebrovascular accident	1.26 (0.80–1.97)	1.30 (0.80–2.10)	0.99 (0.65–1.51)	0.79 (0.50–1.25)	1.36 (0.84–2.21)
Diabetes	0.88 (0.55–1.40)	**1.85 (1.00–3.40)**	0.93 (0.56–1.54)	0.89 (0.45–1.79)	0.79 (0.51–1.21)
Thyroid	0.63 (0.29–1.38)	1.13 (0.58–2.20)	1.22 (0.64–2.36)	1.45 (0.70–3.01)	0.64 (0.28–1.46)
Chronic respiratory	0.73 (0.29–1.88)	1.21 (0.65–2.27)	1.34 (0.81–2.19)	1.11 (0.60–2.07)	1.68 (0.73–3.9)
Cancer	1.23 (0.83–1.83)	1.29 (0.80–2.06)	1.16 (0.78–1.74)	0.92 (0.56–1.50)	0.89 (0.55–1.43)
Parkinson disease	0.55 (0.25–1.21)	1.06 (0.50–2.26)	0.80 (0.49–1.30)	0.75 (0.37–1.53)	1.06 (0.59–1.9)
Depression, mood, and affective disorders	**8.00 (4.98–12.87)**	**5.46 (3.25–9.18)**	**21.24 (13.46–33.51)**	**19.30 (10.90–34.18)**	**2.41 (1.47–3.96)**
Anxiety and stress-related disorders	**2.29 (1.27–4.13)**	**2.50 (1.33–4.68)**	**1.80 (1.12–2.87)**	**3.44 (1.88–6.29)**	**1.78 (1.05–3.02)**
PUD/GORD	**0.69 (0.49–0.97)**	1.04 (0.55–1.97)	0.87 (0.62–1.21)	0.84 (0.51–1.39)	1.02 (0.76–1.38)
Renal disease	1.11 (0.72–1.71)	0.95 (0.60–1.50)	0.95 (0.56–1.62)	0.96 (0.49–1.90)	1.04 (0.71–1.51)
Arthritis	0.95 (0.53–1.69)	0.93 (0.59–1.46)	0.93 (0.61–1.41)	1.05 (0.67–1.66)	0.89 (0.55–1.46)
Osteoporosis	0.68 (0.44–1.05)	1.12 (0.80–1.57)	1.12 (0.73–1.72)	0.86 (0.52–1.41)	0.81 (0.52–1.24)
Gout	0.56 90.21–1.52)	1.83 (0.84–4.03)	1.36 (0.72–2.56)	1.32 (0.45–3.88)	**2.81 (1.19–6.62)**
Fracture	1.13 (0.79–1.61)	1.36 (0.87–2.11)	0.89 (0.53–1.50)	0.80 (0.55–1.16)	1.09 (0.61–1.95)
Hearing impairment	0.86 (0.45–1.67)	0.66 (0.36–1.24)	0.73 (0.44–1.20)	1.21 (0.63–2.34)	1.14 (0.62–2.1)
Visual impairment	0.86 (0.41–1.82)	1.18 (0.56–2.47)	0.82 (0.42–1.58)	0.79 (0.34–1.86)	0.59 (0.27–1.32)
ATC medication classes (Level 1)					
Alimentary tract and metabolism	1.29 (0.62–2.69)	1.54 (0.72–3.26)	0.60 (0.35–1.03)	0.60 (0.30–1.23)	0.86 (0.5–1.46)
Antineoplastic and immunomodulators	1.12 (0.43–2.97)	0.21 (0.02–1.94)	0.72 (0.32–1.64)	1.14 (0.44–2.98)	1.09 (0.41–2.88)
Blood and blood-forming organs	1.00 (0.64–1.57)	0.81 (0.53–1.22)	0.91 (0.62–1.32)	0.71 (0.48–1.04)	0.78 (0.47–1.31)
Cardiovascular system	0.86 (0.61–1.21)	1.35 (0.85–2.16)	1.03 (0.64–1.63)	1.00 (0.62–1.61)	0.78 (0.46–1.32)
Genitourinary system and sex hormones	0.74 (0.40–1.37)	0.50 (0.24–1.06)	0.58 (0.33–1.03)	0.58 (0.28–1.23)	**0.35 (0.16–0.76)**
Musculoskeletal system	1.30 (0.87–1.95)	0.63 (0.32–1.21)	1.06 (0.66–1.72)	0.86 (0.42–1.78)	**0.43 (0.22–0.84)**
Nervous system	**4.70 (2.72–8.11)**	1.37 (0.80–2.36)	**11.57 (5.93–22.55)**	**10.44 (3.02–36.09)**	**4.03 (2.28–7.12)**
Respiratory system	1.06 (0.41–2.75)	1.00 (0.49–2.06)	1.05 (0.65–1.70)	0.85 (0.31–2.37)	0.79 (0.36–1.76)
Sensory organs	1.03 (0.61–1.71)	0.99 (0.56–1.74)	0.90 (0.57–1.42)	0.71 (0.35–1.44)	0.75 (0.49–1.16)
Systemic hormonal preparations	0.70 (0.37–1.31)	0.81 (0.41–1.57)	0.85 (0.50–1.45)	0.75 (0.42–1.33)	0.91 (0.5–1.66)

*Group 1* (psychotropic non-users) as a reference category. *P* < 0.05 is indicated in bold

*Group 2*: Anxiolytics/hypnotics users with gradually rising antidepressant use; *Group 3*: Gradually increasing antidepressant users; *Group 4*: Antidepressant users with negligible exposure to other psychotropics; *Group 5*: Antipsychotic and antidepressant users with low anxiolytics/hypnotics use; *Group 6*: Antipsychotic users with low anxiolytics/hypnotics and negligible antidepressant usage

^**a**^Psychosis was not included in the model due to the small sample size in each group

In the dementia cohort, eleven variables, including four related to medication, were associated with at least one trajectory group (Table 3). Baseline mental health-related diagnoses predicted all groups, while the use of nervous system medications was predictive for all groups except Group 3 in comparison to the reference group. Increasing age was inversely associated with the likelihood of belonging to Group 5 (RRR 0.94; 95% CI 0.91–0.97) or Group 6 (RRR 0.97; 95% CI 0.94–0.99) compared to the reference group. Provider B, compared to Provider A, had a twofold higher relative risk of being in Group 2 (anxiolytics/hypnotics users with gradually rising antidepressant use) over the reference group (RRR 2.02; 95% CI 1.23–3.30). Individuals with diabetes were 1.85 times more likely to be in Group 3* (*gradually increasing antidepressant users) versus the reference group (RRR 1.85, 95% CI 1.00–3.40).

## Discussion

We identified six trajectories of psychotropic medicine use in residents with dementia and four in those without dementia. Our trajectory analysis showed that for all trajectory groups (excluding negligible use of psychotropic medicines in both cohorts) rates of psychotropics use rarely declined over the three-year study period, particularly the rates of antidepressant and antipsychotic use. A high proportion of residents used psychotropic medicine classes concurrently, with approximately one-third of residents with dementia (31.6%) and one in five residents without dementia (23.3%) concurrently using medications from multiple psychotropic classes. We also identified that some factors predicting trajectories were different for people with and without dementia. For example, cardiovascular disease diagnosis at baseline was associated with lower psychotropic use in people without dementia compared to those with dementia.

Our findings highlight potential deviations from current guidelines in the appropriate use of psychotropics in RACFs in several ways. The limited variability in the patterns of their use over time suggests that residents who are using psychotropic medicines often persist with continuous use, rather than gradually tapering after the recommended period, exploring non-pharmacological therapy, and discontinuing them for a period before considering a return if clinically indicated. In the context of dementia, this manifests as a persistent use of antipsychotics and benzodiazepines. Our study revealed that antipsychotic use remained consistent and did not decrease over the three-year period for one in five (20.7%) residents with dementia (Groups *5* and *6*). Australian guidelines recommend no more than 12 weeks of continuous use of antipsychotics for BPSD before reviewing and weaning their use [7]. This pattern of consistent drug use was also evident for antidepressants for all residents. In our study, the rate of antidepressant medicine use rarely showed a reduction in any of the trajectories, which is contrary to Australian guidelines which recommend antidepressant treatment be limited to 6–12 months [7, 23]. Similarly, anxiolytics/hypnotics medicine use was extended beyond the recommended intermittent or two-week window to treat sleep disturbances and panic disorders, in all trajectories, for residents with and without dementia [24, 25]. These findings suggest that the use of psychotropic medicines is persistent in RACF settings and more needs to be done to support deprescribing practices.

Our study revealed a concerning high rate of concurrent use of multiple psychotropic medicine classes in both cohorts (31.6% in dementia and 23.3% in non-dementia cohorts), posing potential risks for residents. Previous Australian studies, using different methods, have also reported high rates of concurrent psychotropic use, ranging from 18 to 26%, in populations with dementia [26, 27]. A small Australian cross-sectional study which reviewed 446 medical charts revealed that 26% of RACF residents received two or more psychotropic medicines [26]. Over the course of a decade (2011–2020), a cross-sectional analysis of Australian general practice data, including 24,701 participants, showed the rate of prescribing of two or more psychotropics in individuals with dementia, ranged from 18.1% to 21.7% [27]. The high rates of concurrent psychotropics use in RACFs could be the result of managing multiple comorbidities or the use of combination therapy by prescribers. Regardless of the underlying cause, this high rate is concerning, posing an increased risk of drug–drug interactions and adverse events [5, 28, 29]. For example, in a cohort study of 3,075 older adults, those who took two or more psychotropic medicines were twice as likely to fall compared to non-psychotropic medicine users [30]. However, it is important to recognise that concurrent psychotropic medicine use is not always inappropriate, as some residents with multiple mental health issues may require various psychotropic medicines for effective management [31]. In the future, prescriber training and further evidence, monitoring, and adherence to guidelines are required to ensure appropriateness of the concurrent use of psychotropics in RACFs.

Our study identified several baseline predictors of trajectory membership. In both cohorts, baseline mental health diagnoses such as depression predicted the type of trajectory residents followed. However, certain variables were exclusive to either dementia or non-dementia cohorts. For instance, a renal disease diagnosis at baseline predicted trajectory membership in the non-dementia cohort, showing an association with lower psychotropic use; however this association was not observed in the dementia cohort. Additionally, a cardiovascular disease diagnosis at baseline was linked to lower psychotropic use in individuals without dementia compared to those with dementia. Identification of such factors in residents’ baseline characteristics associated with persistent psychotropic use may be helpful in prioritising interventions such as residential medication management reviews [32].

### Policy and practice implications

The concurrent use of psychotropics identified in our study has policy and practice implications. Our findings suggest that Australian policy efforts to curb psychotropic medicine use may lack comprehensive coverage. In October 2021 Australia introduced mandatory reporting of antipsychotic medication use in all publicly funded RACFs [33], representing a step to reduce the incidence of chemical restraint in RACFs. However, the reporting of a single psychotropic medicine class may not provide a thorough assessment of potential harm associated with the use of psychotropic medications in this population. This could also lead to a shift in prescribing practices towards other psychotropic classes. For example, in Norway, although the rate of antipsychotic medicine use in RACFs has declined, a longitudinal analysis demonstrated that prescribers turned to alternatives or combinations of alternative medications to manage mental health problems [28]. Our results indicate that concurrent use of psychotropic medicines is already potentially too high. To ensure that antipsychotic substitution is not repeated in Australia, the Quality Indicator Program should consider the monitoring of all psychotropic medicines simultaneously.

### Strengths and limitations

The notable strength of our study lies in its methodology. We employed group-based multi-trajectory modelling, a novel statistical method for modelling multiple indicators over time, utilising a large sample across two aged care providers with 30 facilities. Another strength is our access to medication administration data, providing insights into actual usage, in contrast to the typical focus on prescribed or dispensed medications often used in medication utilisation studies [26, 27]. The main limitation of this study––aligning with general constraints in routinely collected electronic data––is that our analysis relies on the documentation of health conditions at the time of facility admission. Health conditions, including dementia and psychosis-related diagnoses, were recorded as free-text entries rather than structured diagnostic codes. While we used a previously developed health macro to process these entries and identify specific conditions [12], this approach may not capture all relevant cases due to variations in documentation practices and potential inconsistencies in terminology. Additionally, our dataset lacks information about new diagnoses or amended conditions post-admission. This becomes particularly important in the context of a dementia diagnosis, which was the primary variable for our two trajectory models. Consequently, our data may fail to capture new dementia diagnoses over the study period, potentially leading to misclassification bias if residents without dementia at baseline develop dementia post-admission.

## Conclusions

This study offers a comprehensive investigation into the concurrent longitudinal use of three psychotropic medicine classes in RACFs, revealing notable differences in usage patterns for residents with and without dementia. Our novel methodology revealed that approximately one in three residents with dementia and one in five residents without dementia concurrently use multiple psychotropic classes, which may put residents at risk. National monitoring programmes need to go beyond measuring just the proportion of residents using antipsychotics, as this is a blunt instrument for understanding the appropriateness of such use or for targeting strategies for improving care and resident outcomes.

## Data Availability

The data that support the findings of this study are available on request from the corresponding author. The data are not publicly available due to privacy or ethical restrictions.

## Code availability

The code that supports the findings of this study is available upon reasonable request from the corresponding author.
